# PP02 The Application Of Care Pathway Analysis And Economic Modeling In Early Health Technology Assessment: Learnings From Two Projects

**DOI:** 10.1017/S0266462324001764

**Published:** 2025-01-07

**Authors:** Emily Gregg, Karin Butler, Rachael McCool, Sara Graziadio

## Abstract

**Introduction:**

Early health technology assessment (eHTA) can help to explore the potential value of a technology in the early stages of development. Care pathway analysis (CPA) is a method to identify and map clinical decisions in the current and new care pathways (including the new intervention). This work provides examples of applying CPA within the context of eHTA for medical interventions.

**Methods:**

CPA usually involves a pragmatic review to identify and synthesize national/international guidelines that describe the care pathway for the condition of interest. This is typically followed by a qualitative evaluation that can include semistructured interviews with thematic analysis. Interviews with experts are undertaken to understand where (and why) real-world practices differ from published guidance and to validate the care pathway. They also help to evaluate the strengths/weaknesses of the new technology, potential population and role in the pathway, and barriers/facilitators to adoption. The CPA forms the basis of economic modeling that helps assess the monetary value of the new technology.

**Results:**

The application of CPA from two recent projects will be presented: an innovative diagnostic test for respiratory tract infections and a medical device for treating cataracts. Additionally, the value of CPA in eHTA will be described from the technology developers’ perspective. In both projects, CPA was used to inform the potential value propositions of the new technology and its positioning in the care pathway. It also helped to optimize the structure of the early economic model and to identify evidence generation needs. The early model identified the pathway that was more likely to be cost effective in the future.

**Conclusions:**

CPA is a valuable method within the context of eHTA. Alongside identifying the potential role and positioning of the new technology, test developers found the assessment useful for informing internal strategy decisions and discussions with potential external investors. The developers were able to demonstrate the clinical perspective around the value of the test, elicited through an independent and rigorous methodology.

